# An Efficient New Process for the Selective Production of Odd-Chain Carboxylic Acids by Simple Carbon Elongation Using *Megasphaera hexanoica*

**DOI:** 10.1038/s41598-019-48591-6

**Published:** 2019-08-19

**Authors:** Hyunjin Kim, Byoung Seung Jeon, Byoung-In Sang

**Affiliations:** 1 Department of Chemical Engineering, Hanyang University, 222 Wangshimni-ro, Seongdong-gu, Seoul, 04763 Republic of Korea; 2 Centrum for Applied Geosciences, University of Tübingen, Hölderlinstraße 12, 72074 Tübingen, Germany

**Keywords:** Crop waste, Chemical engineering

## Abstract

The caproate-producing bacterium, *Megasphaera hexanoica*, metabolizes fructose to produce C_2_~C_8_ carbon-chain carboxylic acids using various electron acceptors. In particular, odd-chain carboxylic acids (OCCAs) such as valerate (C_5_) and heptanoate (C_7_), were produced at relatively high concentrations upon propionate supplementation. Using a statistical experimental design method, the optimal culture medium was established for the selective production of OCCAs among the total produced acids. In a medium containing 2.42 g L^−1^ sodium acetate and 18.91 g L^−1^ sodium propionate, *M. hexanoica* produced 9.48 g L^−1^ valerate, 2.48 g L^−1^ heptanoate, and 0.12 g L^−1^ caproate. To clarify the metabolism of the exogenous added propionate for OCCAs production, ^13^C tracer experiments were performed by supplementing the culture broth with [1,2,3-^13^C_3_] propionate. The metabolites analysis based on mass spectrometry showed that the propionate was only used to produce valerate and heptanoate without being participated in other metabolic pathways. Furthermore, the carbon elongation pathway in *M. hexanoica* was explained by the finding that the incorporation of propionate and acetate in the produced valerate occurred in only one orientation.

## Introduction

Volatile fatty acids (VFAs) have various applications, such as microbial nitrogen removal, the production of biofuels, the generation of electricity using microbial fuel cells, the synthesis of biosurfactants and biopolymers, and usages in the food, textile, pharmaceutical, leather, and plastics industries^1–7^. VFAs that are secreted extracellularly can be classified into short-chain carboxylic acids (SCCAs), such as acetic acid, propionic acid, and butyric acid, and medium-chain carboxylic acids (MCCAs) with chains of 5~8 carbons (valeric acid, caproic acid, heptanoic acid, and caprylic acid). During anaerobic fermentation, SCCAs are elongated to MCCAs by the addition of 2-carbon (C_2_) units. Several bacteria have been reported to produce MCCAs by the reverse β-oxidation pathway (r-BOP)^8,9^.

Generally, the value of VFAs increases with the increasing carbon number. The market prices of different VFAs increase in the following order: acetic acid (400 ~ 700 USD/ton), propionic acid (1,900 ~ 2,000 USD/ton), butyric acid (2,000 ~ 2,200 USD/ton), and valeric acid (2,500 ~ 3,000 USD/ton). This pricing pattern can be attributed both to the wider industrial application of longer-chain carboxylic acids and to the greater difficulty of their production than that of SCCAs^7^. Therefore, there are many research reports on the r-BOP in which SCCAs are elongated to MCCAs, which have a higher economic value and are easier to separate and purify^10–13^. To convert SCCAs into MCCAs via condensation with acetyl-CoA by the r-BOP, energy-rich reduced compounds are needed to provide energy and reduce equivalents. Acetyl-CoA adds two carbons in each subsequent round of fatty acid synthesis. Consequently, acetate (C_2_) can be elongated to the butyrate (C_4_) and further elongated to caproate (C_6_) and caprylate (C_8_). Odd-carbon chains can also be produced in the presence of high cellular propionyl-CoA levels due to their incorporation in place of acetyl-CoA in the initial step of fatty acid synthesis. In this case, propionate (C_3_) will be elongated to valerate (C_5_) and further elongated to heptanoate (C_7_). Since acetyl-CoA is a common natural biogenic precursor for the biosynthesis of numerous metabolites, while propionyl-CoA is toxic at high concentrations, propionyl-CoA is rare and non-native to most organisms^14^. Presumably, most organisms have evolved to prevent the intracellular accumulation of propionyl-CoA^14^. Therefore, various studies have been conducted on even-chain MCCA production, but the production of odd-chain MCCAs, which are industrially useful as plasticizers and herbicides, as well as in the fragrance industry, has not been actively investigated^15–17^. Odd-chain carboxylic acid (OCCA) production has previously been achieved, but the concentration of OCCAs was low, and their productivities were lower than those of even-chain carboxylic acids (ECCAs)^18,19^.

Various chain elongation processes have been proposed to obtain OCCAs, such as valerate and heptanoate. A reactor microbiome consisting of a continuously stirred tank reactor (CSTR) supplied with propionate and ethanol produced 4.6 g L^−1^ valerate, 4.9 g L^−1^ caproate and 3.2 g L^−1^ heptanoate^18^. Another CSTR that utilized the initial sludge inoculum that was obtained from a full-scale anaerobic digester treating potato waste had a main elongated product of 3.1 g L^−1^ valerate along with 1.2 g L^−1^ caproate and traces of heptanoate from the propionate−ethanol mixture^19^. A pure culture of *Clostridium kluyveri*, which was also supplied with propionate and ethanol, produced 7.4 g L^−1^ valerate in a batch reactor after 18 days^20^, in addition to 0.8 g L^−1^ caproate and 0.3 g L^−1^ heptanoate. In contrast to mixed-culture fermentation, although the productivity was very low, the pure culture of *C. kluyveri* showed high selectivity for valerate. In another example, *Megasphaera hexanoica*, which was isolated by our group, produced various MCCAs, including both even- and odd-chain carboxylic acids (C_5_~C_8_) with high productivities and titers when C_2_, C_3_, and C_4_ carboxylic acids were added to the medium as electron acceptors^9,21^. In a medium containing acetate and propionate with fructose, *M. hexanoica* generated 5.7 g L^−1^ valerate, 1.5 g L^−1^ caproate, and 2.7 g L^−1^ heptanoate within just 1 day, showing a high selectivity of 85% for OCCAs. These physiological characteristics of *M. hexanoica* are expected to enable a process to generate OCCAs with high selectivity and productivity. Since anaerobic digestion of organic wastes has recently gained attention as a cost-effective and environmentally friendly alternative to acetate and propionate production through the prevailing acidogenic metabolic pathways in the digester, for the industrial implementation of OCCA production by *M. hexanoica*, the acetate and propionate from the anaerobic digestion process can be attractive and promising SCCAs.

Jeon *et al*. showed that the production of ECCAs was influenced by the concentrations of acetate and butyrate, and the response surface method (RSM) experiments were conducted to get an optimized medium composition, including acetate and butyrate for *Caproiciproducens galactitolivorans*, as well as *M. hexanoica*^22,23^. Adding butyrate into the medium could stimulate the production of ECCAs. Therefore, experiments tracing the ^13^C isotope were conducted to explain the direct conversion of the supplemented acids, such as acetate and propionate, into OCCAs.

The objective of this study was to achieve efficient production of OCCAs, such as valerate and heptanoate, with a high selectivity and production rate throughout the r-BOP with supplemented SCCAs by *M. hexanoica*. Then, the carbon elongation mechanism for the production of OCCAs by *M. hexanoica* was investigated in tracking experiments that involve ^13^C-labelled propionic acid. The findings from this study demonstrate that through the application of selective operating conditions for OCCA production, optimized compositions of supplemented SCCAs and extractive fermentation can be reproducibly driven towards the simultaneous stable production of high levels of MCCAs, especially specific OCCAs with higher levels of SCCAs conversion.

## Results

### OCCA production by *M. hexanoica* with supplemented propionate

*M. hexanoica* has been reported to produce various OCCAs, including valeric acid and heptanoic acid, to 4.1 g L^−1^ and 2.0 g L^−1^ in a medium that contains supplemented propionate^9^. Along with the change of propionate by the supplemented amount, the adjustment of acetate concentration was selected as a significant factor, because acetate has been reported to be an electron acceptor that increases cell growth and metabolite production in *Megasphaera* species^24^. Jeon *et al*. showed that the addition of acetate along with propionate increased the concentration of valeric acid and heptanoic acid by 1.3~1.4 times^9^. However, as shown in the Supplementary Figure S1, the addition of excess acetate reduced the selectivity of OCCAs. Therefore, the optimum concentration of acetate with propionate needs to be determined. Until now, no optimized conditions for OCCA production with supplemented SCCAs had been developed, and the factors that affect the propionate-based carbon elongation mechanism via r-BOP had not been investigated in depth.

### Verification of OCCA production at the optimized medium compositions

To investigate the production of OCCAs from the supplemented SCCAs (acetate and propionate), the ranges of two independent variables (i.e., the concentrations of sodium acetate and sodium propionate) were decided according to the results of a series of experiments; namely, the one-fact-at-a-time test (OFAT), the fractional factorial experimental design (FFD), and the steepest ascent method experimental design (SAM)^22,25,26^. From these analyses, the concentration ranges of sodium acetate and sodium propionate were set to 2.29–2.51 g L^−1^ and 16.46–23.54 g L^−1^, respectively. To clarify the optimized concentration of supplemented acetate and propionate, an investigation using response surface methodology (RSM) with central composite design (CCD) was conducted. The valerate production was selected as the response due to the different cycles of the runs. The experimental design matrix was presented in Table 1. Thirteen experiments were performed in duplicate. A regression model was fitted to the production of valeric acid and was obtained in the form of the following equation:

1 $${\rm{Valeric}}\,{\rm{acid}}\,{\rm{production}}\,(g\,{{\rm{L}}}^{-{\rm{1}}})={\rm{12.55}}-{\rm{2}}{{\rm{.66X}}}_{{\rm{2}}}-{\rm{2}}{{\rm{.95X}}}_{2}^{2}$$

where X_2_ represents the coded value of sodium propionate.

**Table 1:** Central composite experimental design matrix and experimental responses.

Run	Real value level (g L^−1^)		Coded value		Valeric acid (g L^−1^)
	Sodium acetate	Sodium propionate	X_1_	X_2_	
1	2.32	17.50	−1	−1	9.99
2	2.48	17.50	1	−1	10.23
3	2.32	22.50	−1	1	5.34
4	2.48	22.50	1	1	7.18
5	2.29	20.00	−1.41	0	11.86
6	2.51	20.00	1.41	0	12.74
7	2.40	16.46	0	−1.41	12.28
8	2.40	23.54	0	1.41	2.65
9	2.40	20.00	0	0	11.38
10	2.40	20.00	0	0	12.06
11	2.40	20.00	0	0	13.29
12	2.40	20.00	0	0	12.58
13	2.40	20.00	0	0	13.34

**Figure 1:**
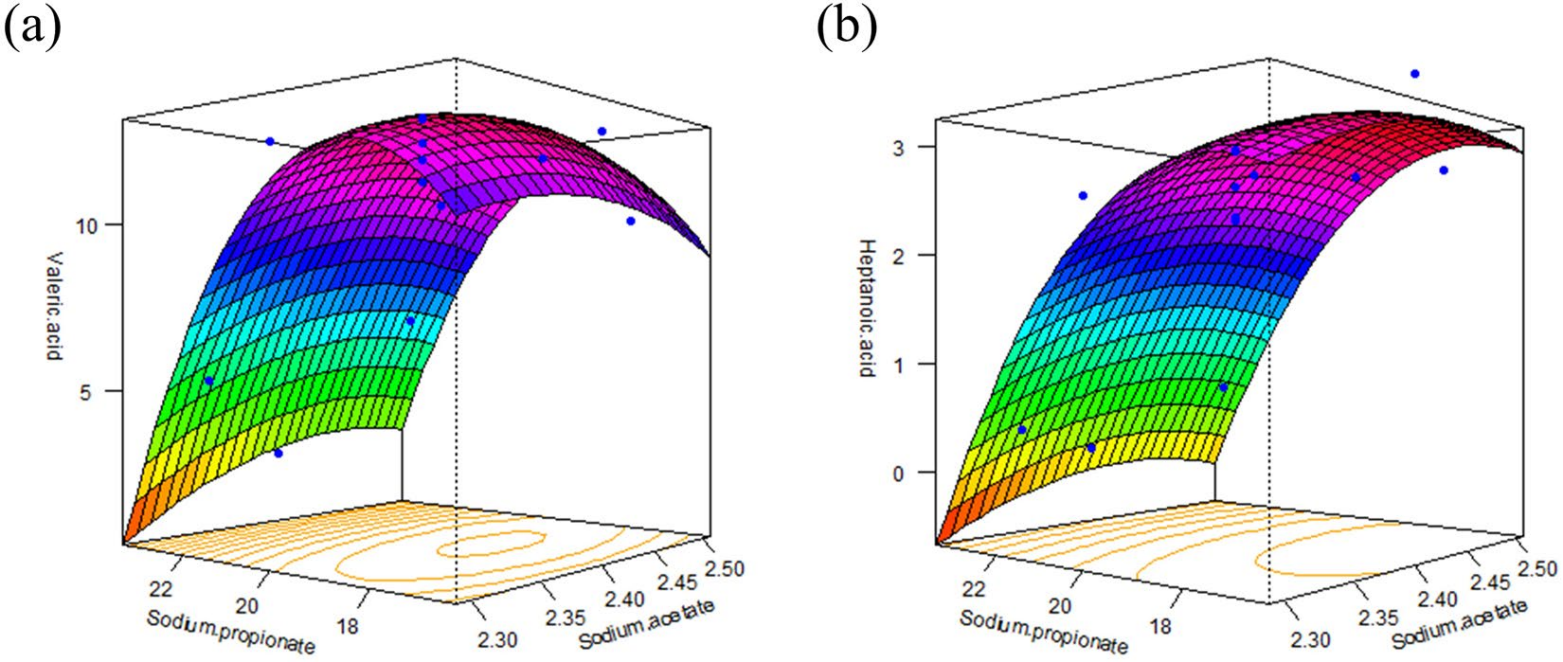
Predicted model: 3D contour plot showing the effect of the amounts of sodium acetate and sodium propionate on the production of (**a**) valeric acid and (**b**) heptanoic acid by *Megasphaera hexanoica*. Blue points show actual value.

Using an analysis of variance (ANOVA) to evaluate the appropriateness of the model, the predicted model was examined by an F-test and the coefficient of determination, R^2^. The F and p values of the model were 13.11 and 0.0019, respectively (Table 2). R^2^ and the adjusted R^2^ were 0.90 and 0.83, respectively, which indicated that the regression model is appropriate for describing the results that are presented in Table 2. As shown in Equation (1), the p values of X_2_ and X_2_^2^ were significant, i.e., p < 0.05 (Table 2), but X_1_, X_1_^2^, and X_1_X_2_ had p > 0.05, which indicated that they were not significant. This result means that the concentration of sodium acetate did not significantly influence valeric acid production; therefore, sodium acetate (X_1_) was excluded from the regression model equation. From the optimum point for valeric acid production, which is shown in Fig. 1a, the optimized medium compositions were 2.42 g L^−1^ of sodium acetate and 18.91 g L^−1^ of sodium propionate, respectively. In the optimized compositions, the maximized concentration of valeric acid was calculated to be 13.15 g L^−1^ (Fig. 1a). In addition, as shown in Fig. 1b, heptanoic acid production was also calculated in the 3D contour plot, with an optimum point for heptanoic acid production (see Supplementary Information, Tables S1, S2 and Eq. S1). When the sodium acetate and sodium propionate were 2.44 g L^−1^ and 17.64 g L^−1^, respectively, the model predicted 3.21 g L^−1^ of heptanoic acid. The optimum conditions for heptanoic acid overlapped with the optimum range for valeric acid production, which indicated that heptanoic acid production was closely related to valeric acid production.

**Table 2:** Results of the statistical analysis of the central composite experimental design for valeric acid production.

	Coefficient estimate	*t*-Value	*p*-Value
Intercept	12.55	20.74	<0.0001
X_1_	0.42	0.87	0.4123
X_2_	−2.66	−5.57	0.0008
X_1_X_2_	0.40	0.59	0.5730
X_1_^2^	−0.59	−1.11	0.3042
X_2_^2^	−2.95	−5.76	0.0007
		**F-Value**	***p*** **-Value**
Model		13.11	0.0019
Lack of fit		4.81	0.0815

*R*^2^ = 90.35%, *R*^2^ (adjusted) = 83.46%.

**Figure 2:**
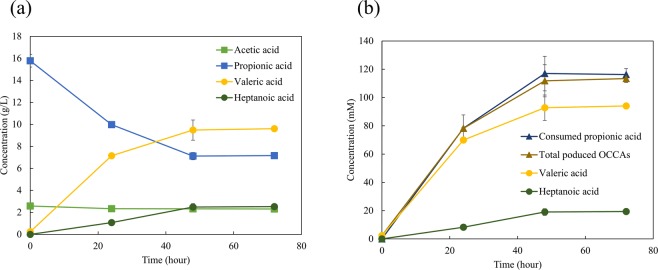
Time course of carboxylic acids concentration in the optimized medium by *Megasphaera hexanoica* expressed in (**a**) mass concentration and (**b**) molar concentration.

To verify the optimum conditions for OCCA production, the optimum conditions were evaluated by a time course experiment in a batch reactor. The results showed that the predicted value was similar to the experimental values (Fig. 2a). *M. hexanoica* produced 9.48 g L^−1^ valeric acid and 2.48 g L^−1^ heptanoic acid when grown in the optimized medium composition for 48 h. Interestingly, the acetate concentration was unchanged during fermentation because the produced acetate by *M. hexanoica* using fructose was utilized on the OCCAs production with supplemented propionate, and the amounts of even-carbon MCCAs, such as caproic acid and butyric acid, were extremely low. The produced amounts of OCCAs were 99% of the total produced MCCAs, with a highly selective production of OCCAs. The productivities of valerate in this study were higher than that of any other previous results and were almost two times higher due to extractive fermentation with a fed-batch culture (Table 3).

**Table 3:** Performance comparison of biological fatty acid (FA) production, OCCA selectivity, and valerate productivity.

Strains	A	B	C	D	E	F	G
	*Megasphaera hexanoica*	*Megasphaera hexanoica*	*Megasphaera hexanoica*	*Megasphaera hexanoica*	*Clostridium kluyveri*	Microbiome	Microbiome
Substrate	Fructose + acetate + propionate	Fructose + acetate + propionate	Fructose + propionate	Fructose + acetate + propionate	Ethanol + propionate	Ethanol + propionate	Ethanol + acetate + propionate
Fermentation mode	Batch	Fed-batch with product extraction	Batch	Batch	Batch	CSTR	CSTR
Fermentation time (day)	2	4	1	1	18	9	21
**Reactor concentration**							
Valerate^a)^ (g L^−1^)	9.5	12.4	4.1	5.7	7.4	3.1	4.6
Caproate (g L^−1^)	0.1	0.4	0.2	1.5	0.8	1.4	4.9
Heptanoate (g L^−1^)	2.5	(Not measured)	2	2.7	0.3	0.4	3.2
Sum total of FAs ≥ 5 C-atoms	12.1	12.4	6.3	9.9	8.5	4.9	12.7
**Selectivity**							
OCCAs^b)^ (mol mol^−1^ × 100%)	99	97	97	86	92	74	62
**Productivity**							
Valerate^c)^ (g L^−1^ h^−1^)	0.34	0.51	0.17	0.24	0.02	0.01	0.27
Reference	This study	This study	Jeon *et al*.^9^	Jeon *et al*.^9^	Bornstein and Barker^20^	Coma *et al*.^19^	Grootscholten *et al*.^18^

^a)^In CSTR reactors, the concentration of acids was measured at steady state.

^b)^The selectivity is defined as the molar concentration of odd-numbered acids divided by the net acid production.

$$\frac{{C}_{valerate}+{C}_{heptanoate}}{{C}_{valerate}+{C}_{caproate}+{C}_{heptanoate}}$$

^c)^In CSTR reactors, the productivity was calculated by dividing the product concentration by HRT.

*M. hexanoica* has been reported to produce even-carbon MCCAs, mainly caproic acid, up to 9.7 g L^−1^ with fructose as the sole carbon source^9^. Even in our case, *M. hexanoica* consumed 20 g L^−1^ fructose as a carbon source, but most of the produced MCCAs were extremely biased towards OCCAs. Therefore, an important question was whether the addition of propionic acid stimulated OCCA production or it was self-incorporated. In particular, Fig. 2b shows that the supplemented propionate was utilized to produce OCCAs with high selectivity in comparison with the consumed and produced molar concentrations of each compound.

### Investigation of selective OCCA production with carbon elongation mechanisms by r-BOP using supplemented ^13^C propionate

**Figure 3:**
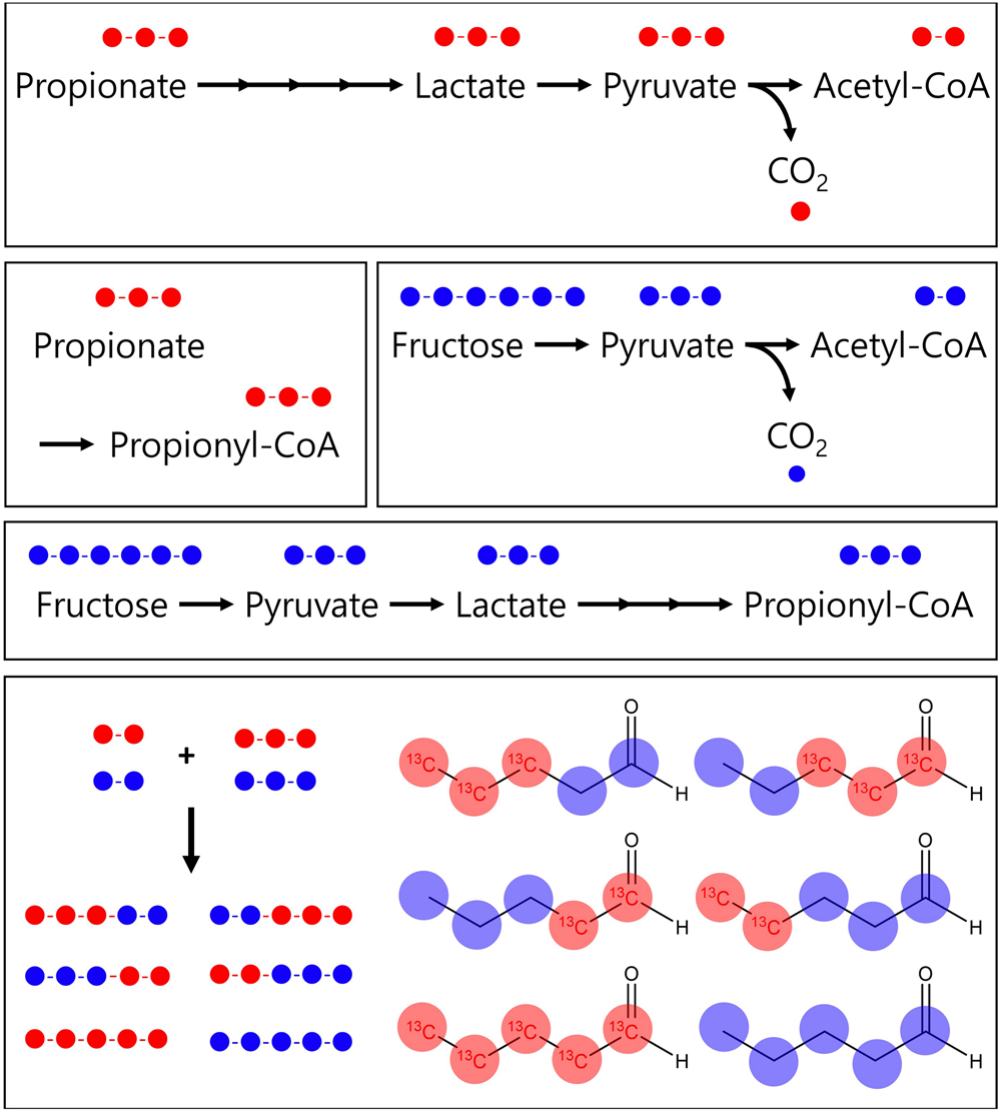
Predicted pathways to produce acetyl-CoA and propionyl-CoA as building blocks of valeric acid and possible forms of valeric acid. Labelled ^13^C atoms, red circles; ^12^C atoms, blue circles.

Propionate labelled with ^13^C for all carbons was supplemented to trace the position of the propionate incorporation into produced MCCAs and to investigate carbon elongation through r-BOP under the optimal condition mentioned above. Predecessor tracking of the ^13^C isotope was conducted to eliminate the possibility of the decomposition of propionic acid. As shown in Fig. 3, through the acrylate pathway, acetyl-CoA can be generated with one CO_2_ per molecule from extracellular propionate. The mass spectra of all fatty acids, including even-carbon fatty acids, were scrutinized. However, ^13^C isotopes were observed only in OCCAs, such as valeric acid and heptanoic acid (see Supplementary Information, Fig. S2). In addition, the analysis of gas components, such as CO_2_ and H_2_ by GC-MS, did not detect CO_2_ containing the ^13^C isotope in the gas phase (see Supplementary Information, Fig. S3).

**Figure 4:**
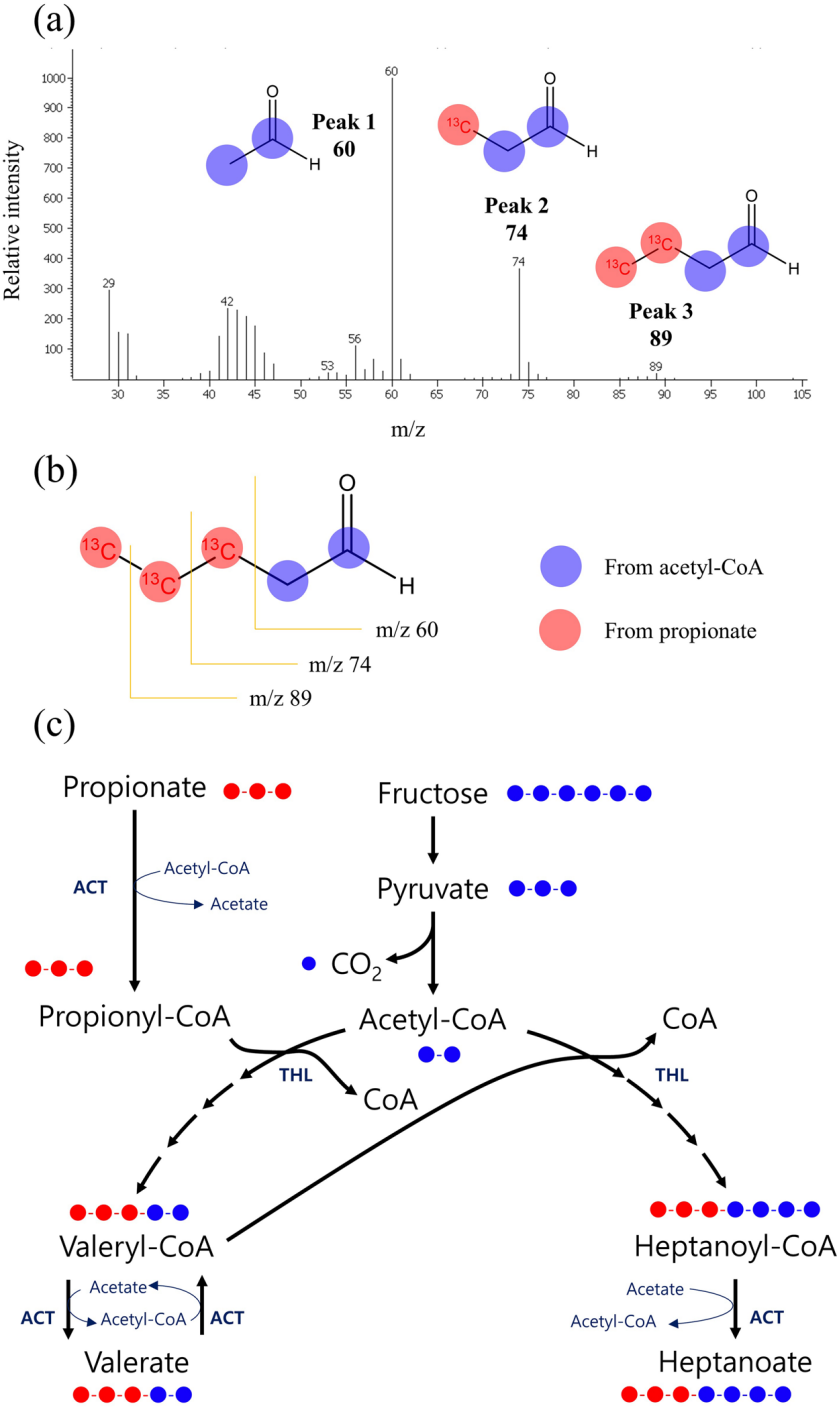
Mass spectrum of valeric acid obtained by GC-TOF/MS and the predicted structure of produced valeric acid. (**a**) Mass spectrum of valeric acid in a culture broth of *Megasphaera hexanoica* with [1,2,3-^13^C_3_] propionate. (**b**) Predicted structure of the resulting valeric acid with ^13^C-labelled moieties at the 3^rd^ to 5^th^ carbons. (**c**) Metabolic pathways of fractional ^13^C-labelled intermediate metabolites produced from [1,2,3-^13^C_3_] propionate input by *Megasphaera hexanoica*. Labelled ^13^C atoms, red circles; ^12^C atoms, blue circles.

The mass spectrum for the produced valeric acid was shown in Fig. 4. In Fig. 4a, the first ion, m/z 60 (peak 1), was observed, which indicated that the m/z 60 ion contained a stable isotope and was derived from carbohydrates, such as fructose. However, the m/z 73 ion (^12^C_3_H_6_O_2_^+^) and the m/z 87 ion (^12^C_4_H_8_O_2_^+^) were shifted to m/z 74 (peak 2, ^13^C^12^C_2_H_6_O_2_^+^) and to m/z 89 (peak 3, ^13^C_2_^12^C_2_H_8_O_2_^+^), respectively, which indicated ^13^C labelling at the 3^rd^ and 4^th^ carbons of the produced valeric acid from the supplemented ^13^C-labelled propionic acid (Fig. 4b). The ^13^C isotope was also incorporated at the 5^th^ carbon of valeric acid, since the mass fragment at m/z 104 appeared faintly. This result indicated that extracellular propionic acid was incorporated entirely into >C5 OCCAs, without decomposition by any catabolic pathway, and the ^13^C isotopes were observed at the 3^rd^, 4^th^ and 5^th^ carbons of valeric acid (Fig. 4b). In the genomic analysis of *M. hexanoica* (data not shown), there is no metabolic pathway for propionate utilization (including the acrylic pathway) in *M. hexanoica*. Thus, the extracellular propionic acid was only used as an electron acceptor via the r-BOX pathway for OCCAs production.

## Discussion

The supplemented SCCAs generally can be elongated to MCCAs through two different metabolic pathways, the keto-acid pathway (KAP) and the r-BOP. The engineered r-BOP showed better performance than KAP in synthesizing the MCCAs of varying carbon lengths from the supplemented SCCAs. Although the r-BOP can convert the SCCAs to MCCAs at a faster rate with low energy consumption, previous reports showed two bottleneck steps of the r-BOP, such as acyl-CoA synthetase (or transferase) and thiolase activities^27^. In the engineered r-BOP for pentanol (C5-alcohol) synthesis in engineered *E. coli*, pentanol was observed as only a minor component in the product mixture, and propionate supplementation increased the pentanol production^28^. To enhance the selectivity and to accelerate the productivity of the desired MCCA products, the information about bottleneck nodes in r-BOP should be updated, as this would facilitate further metabolic engineering.

Recently, MCCAs as secondary products of anaerobic fermentation via the r-BOP have been reported^7^ in anaerobic bioreactors that were inoculated with open cultures and fed with diverse carbon sources, such as ethanol, organic wastes, and sugars^10,29,30^. The microbiome in the anaerobic bioreactors showed that *Megasphaera* species play an important role as MCCAs producers^30,31^. In particular, *Megasphaera elsdenii*^24^ and *Megasphaera indica*^32^ showed excellent capability to produce MCCAs through chain elongation of the r-BOP using supplemented SCCAs. Recently, the production of valeric acid from the supplemented propionate by *M. elsdenii*^33^ demonstrated that *Megasphaera* species can produce not only ECCAs but also OCCAs. In this study, detailed carbon elongation mechanisms for the OCCA production of our newly isolated *M. hexanoica* was investigated using ^13^C labelled propionic acid.

In our previous study, *M. hexanoica* produced 5.7 g L^−1^ of valeric acid in medium supplemented with 100 mM acetate and propionate for OCCA production^9^. Bornstein and Barker reported selective OCCA production for up to 91% of the total MCCAs produced by *C. kluyveri* from the supplemented ethanol and propionate^20^. However, the batch culture was operated for 18 days until 7.4 g L^−1^ of valeric acid was obtained (Table 3). Likewise, Steinbüsch *et al*. reported heptanoate production from ethanol and propionate via carbon elongation with the r-BOP^18^. However, the selectivity of OCCAs was relatively low (Table 3), because the formation of acetyl-CoA from the supplemented ethanol was preferred to the propionyl-CoA formation from supplemented propionate^18^. In this study, using RSM, the optimum condition for highly selective OCCA production was obtained. Under the optimized condition, *M. hexanoica* produced 9.48 g L^−1^ of valeric acid and 2.48 g L^−1^ of heptanoic acid, while the selectivity of OCCAs was 99% (Table 3). In particular, the valeric acid concentration was up to 12.4 g L^−1^ with 0.51 g L^−1^ h^−1^ of productivity from the extractive fermentation with fed-batch culture. To our knowledge, this resulted in the highest production and productivity of valeric acid using anaerobic fermentation.

The molar concentration of consumed propionic acid was compared to the molar concentration of produced OCCAs: 117.0 ± 12.3 mM propanoic acid was consumed, and 109.5 ± 11.3 mM OCCAs (90.5 ± 8.9 mM of valeric acid and 19.0 ± 2.4 mM of heptanoic acid) was produced (Fig. 2b). As a result, the molar concentrations of acid consumed and produced were similar, which implied that the added propionic acid was incorporated into OCCAs. Thus, to understand what mechanism is used to convert propionate into valerate in *M. hexanoica*, tracking experiments using ^13^C labelled propionic acid ([1,2,3-^13^C_3_] propionate) were conducted. The tracking experiment using ^13^C isotope was an excellent, effective analysis tool of ABE fermentation^34,35^ and the acrylate pathway of *M. elsdenii*^36^.

Performing the tracking experiment using [1,2,3-^13^C_3_] propionate, it was revealed that the propionate incorporated into valerate without decomposition and the ^13^C isotopes were observed at the 3^rd^, 4^th^ and 5^th^ carbons of the valeric acid. The incorporation mechanism of propionic acid seemed to coincide with a carbon elongation mechanism using the r-BOP. The r-BOP tends to increase the carbon number of carboxylic acids by two carbons. The postulated incorporation mechanism, as shown in Fig. 4c, is that one valeric acid could be condensed from propionyl-CoA and acetyl-CoA. Similarly, valeryl-CoA could be condensed with one acetyl-CoA molecule given to heptanoyl-CoA. Although the heptanoic acid produced by the ^13^C-labelled propionic acid would not be predicted as a single isotope sequence in the GC-TOF/MS spectrum, if the reaction proceeded according to the r-BOP, then the heptanoic acid incorporating the added propionic acid, as shown in Fig. 4c, would have ^13^C located at the 5^th^, 6^th^ and 7^th^ carbons.

The r-BOP in this study started with the extracellular propionate entry into the cell. Some anaerobic bacteria, such as *Clostridium actobutylicum* ATCC 824, take extracellular carboxylic acids into the cell by phosphotransbutyrylase (Ptb) and butyrate kinase (Buk)^37^. The reaction mediated by Ptb-Buk produces or consumes ATP. On the other hand, acetyl-CoA transferase (ACT) mediates the uptake and secretion reaction without ATP. With regard to the secretion, ACT was predicted as an enzyme that catalyses the reaction to form caproate from caproyl-CoA in the putative pathway of caproic acid production in *Ruminococcaceae* bacterium CPB6 and *M. elsdenii*^38,39^. The enzyme was expected to catalyse in the reverse direction. In a similar reaction, propionyl-CoA transferase (PCT) from *M. elsdenii* mediates the reaction to form propionyl-CoA from the extracellular propionate^40^. Tseng *et al*. produced odd-chain fuels and chemicals via genetic engineering *Escherichia coli* inserted a *pct* gene from *M. elsdenii*^28^. It is predicted that ACT plays the role of introducing the extracellular carboxylic acid into the cell in *M. hexanoica*, as well. Indeed, a caproic acid-producing *E. coli* strain was constructed by expressing genes encoding ACT from *M. hexanoica*^41^. Thus, the carboxylic acids entering into the cell were converted and used in acyl-CoAs form throughout the r-BOP for acyl-CoA chain elongation. Thiolase (Thl) is involved in the condensation reaction between acetyl-CoA with acyl-CoA modules. In conventional genetic engineering to produce butyric acid or butanol, thiolase genes, such as AtoB from *E. coli*, were used to condense two acetyl-CoA molecules^42^. BktB from *Ralstonia eutropha* H16, another type of thiolase, is involved in the biosynthesis of longer chain polymers. BktB catalyses not only a condensation reaction between two acetyl-CoA molecules to produce acetoacetyl-CoA, but it also catalyses a condensation reaction between acetyl-CoA and propionyl-CoA to produce valeryl-CoA^43^. Furthermore, BktB could catalyse a condensation reaction between acetyl-CoA and butyryl-CoA to form 3-ketocaproyl-CoA, which can be used to produce caproate or n-hexanol (C6-alcohol)^44^. Although BktB showed a slow conversion rate of propionyl-CoA and butyryl-CoA into longer carbon substances, the protein crystal structure analysis of BktB revealed that the residues of amino acids around its active site form the proper structure for longer carbon chain products^45^.

In this study, *M. hexanoica* showed excellent selectivity and productivity of OCCA production using supplemented SCCAs. Through a tracking experiment using ^13^C isotope, the metabolic pathway of producing OCCAs in *M. hexanoica* was proposed as the r-BOP, including ACT and Thl as key enzymes. Thus, its excellent selectivity and productivity might be closely related to the excellent catalytic potential of ACT and Thl in *M. hexanoica*. More detailed information on ACT and Thl of *M. hexanoica* will be available after the completion of our investigation, which is current being conducting.

## Methods

### Media and culture conditions

All bacterial cultures were performed in an anaerobic environment. *Megasphaera hexanoica* was cultivated in a mPYF medium, which contained the following components dissolved in distilled water to a final volume of 1 L: yeast extract, 10 g; peptone, 5 g; tryptone, 5 g; beef extract, 5 g; fructose, 20 g; K_2_HPO_4_, 2 g; cysteine HCl·H_2_O, 0.5 g; hemin solution, 10 mL; and salt solution, 40 mL. The salt solution was prepared in distilled water to a final volume of 1 L: CaCl_2_·2H_2_O, 0.25 g; MgSO_4_·7H_2_O, 0.5 g; K_2_HPO_4_, 1 g; KH_2_PO_4_, 1 g; NaHCO_3_, 10 g; and NaCl, 2 g. To prepare the hemin solution, 50 mg of hemin (Sigma Aldrich) was dissolved in 1 mL of 1 N NaOH and then was diluted in distilled water to a final volume of 100 mL.

For the RSM experiments, sodium acetate and sodium propionate were used as factors and valeric acid was used as a response to maximize the production of valeric acid. Sodium acetate and sodium propionate were added to liquid broth before autoclaving. The liquid broth was prepared as 20 mL of medium in a 60 mL serum bottle under argon purging.

For extractive fermentation, a mixture of solvent solution of 10% (v/v) alamine 336 (Cognis) in oleyl alcohol (Ecogreen, DHW) was used. The solvent solution was washed by distilled water to remove trace elements and unknown chemicals that may inhibit the growth of *M. hexanoica* dissolved in an aqueous medium. Then, the mixture was autoclaved at 121 °C for 15 min before being used for the product extraction. The experiment was conducted in a 3-L fermenter (Fermentec FMT ST, Korea) with 1 L solvent solution. The seeding (5%, v/v) in the mid-exponential phase was inoculated into 1 L medium supplemented with optimum concentration of acetate and propionate. It was cultured for one day without extractive solvent to prevent the toxic effects of the solvent against bacterial cells. After one day of cultivation, the solvent solution was injected by a peristaltic pump onto the cultured broth. The biphasic status of the solvent and the aqueous culture of the broth was maintained during the extractive fermentation in the fed-batch culture, even though the fermenter was stirred at 80 rpm. As needed, the concentrated medium (10× nutrients along with 1× salts and concentrated fructose), was fed into the reactor to bring the fructose concentration up to 30 g/L and to provide nutrients for the culture.

For the isotope analysis, liquid broth was prepared as 2 mL media in a 16 × 125 mm Hungate tube under argon purging. Propionic acid with ^13^C at all three carbons (CLM-647, Cambridge Isotope Laboratories, Inc) was used at the optimal concentration as determined by the RSM experiments.

The pH of the medium was adjusted to 6.5 using 1 N HCl. Bacteria were cultured in a shaking incubator with rotation at 150 rpm and 37 °C. For this purpose, 5% (v/v) seed culture in mPYF was inoculated into a fresh mPYF medium. All experiments were performed in duplicate, and the results are shown as an average of the duplicate experiments.

### Experimental design

The data from the fractional factorial experimental design were used for further analysis with the method of steepest ascent. The method of steepest ascent is a procedure for sequentially moving along the steepest ascent path, which was used to obtain the concentration ranges of variables for a central composite experimental design. A two-factor CCD consisting of 13 experimental runs with five replications at the central point was used to optimize the independent variables. The experimental results obtained with the central composite model were statistically analysed for reliability by ANOVA in R (version 3.2.3), by using the package ‘rsm’ to standardize the acetate and propionate the treatment axes^46,47^.

### Analytical methods

The medium optimization experiments were conducted in duplicate, and the means are shown with standard deviations presented by error bars. The fatty acids in the broth were analysed by a gas chromatogram (GC; Agilent 7890, USA) equipped with a flame ionization detector, according to a previously described procedure^48^.

For isotope analysis in the gas phase, GC-MS was carried out with an Agilent 5975MS coupled with an Agilent 6890GC equipped with an HP-1MS column (60 m × 0.25 mm; film thickness 0.25 µm). The inlet temperature was 150 °C. The initial oven temperature was 40 °C, which was then increased to 0.5 °C/min to 45 °C, held for 4 min, and then was increased at 30 °C/min to 200 °C. The MS transfer line was held at 250 °C.

The GC-MS was performed on a TOF-MS (Leco Pegasus III) coupled with an Agilent 6890GC equipped with an HP-FFAP column (50 m × 0.2 mm; film thickness 0.33 µm) for isotope analysis in the liquid phase. The inlet temperature was 160 °C. The initial oven temperature of 130 °C was increased at 2 °C/min to 180 °C, then at 10 °C/min to 210 °C, and held for 2 min. The MS transfer line was maintained at 240 °C.

## Supplementary information

SUPPLEMENTARY INFORMATION
