# Supplementary material for: An Efficient New Process for the Selective Production of Odd-Chain Carboxylic Acids by Simple Carbon Elongation Using Megasphaera hexanoica
Source: Sci Rep. 2019 Aug 19;9:11999. doi: 10.1038/s41598-019-48591-6 (PMC6700076; doi:10.1038/s41598-019-48591-6)
Supplement: Supplementary file 1 — SUPPLEMENTARY INFORMATION [file 41598_2019_48591_MOESM1_ESM.docx]

SUPPLEMENTARY INFORMATION

**An Efficient New Process for the Selective Production of Odd-Chain Carboxylic Acids by Simple Carbon Elongation Using *Megasphaera hexanoica***

Hyunjin Kim, Byoung Seung Jeon, and Byoung-In Sang^*^

Affiliation:

Department of Chemical Engineering, Hanyang University, 222 Wangsimni-ro, Seongdong-gu, Seoul 04763, South Korea

^*^Corresponding Author:

Dr. Byoung-In Sang, Department of Chemical Engineering, Hanyang University, 222 Wangsimni-ro, Seongdong-gu, Seoul 04763, South Korea

Tel.: +82. 2. 2220. 2328, Fax: +82. 2. 2220. 4716, E-mail: [biosang@hanyang.ac.kr](mailto:biosang@hanyang.ac.kr)

**Fig. S1.** Selectivity comparison for OCCAs produced by addition of propionate and propionate + acetate. Upper diagram indicated selectivity by propionate and lower indicated selectivity by propionate + acetate. The selectivity was re-calculated from the result of Jeon *et al.* 2016 [^9^](#_ENREF_9)

**Fig. S2.** The GC-TOF/MS spectra of *Megasphaera hexanoica* in culture broth with [1,2,3-^13^C_3_] propionate. (a), Acetic acid; (b), butyric acid; (c), caproic acid; and (d), heptanoic acid. The upper right spectra are the library data. The ^13^C-atom was not found in any of the even-numbered acids.

(a)


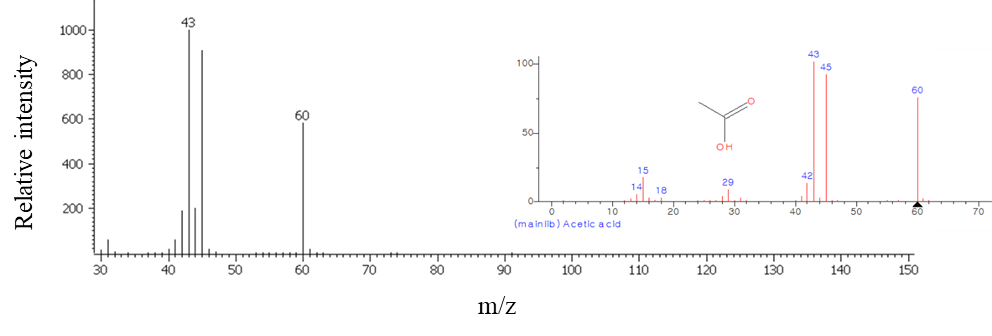


(b)


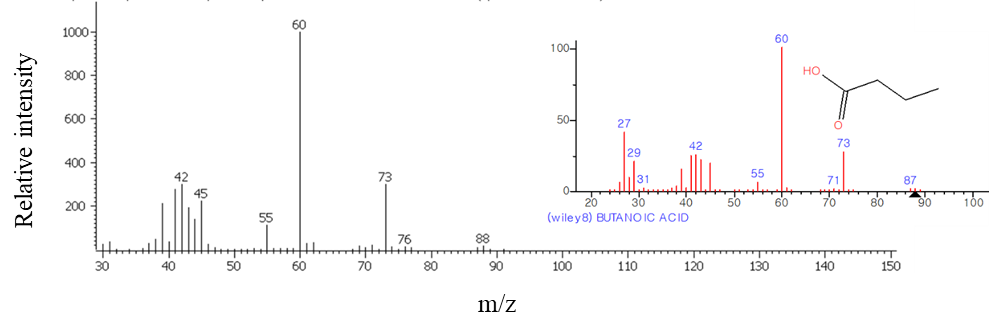


(c)


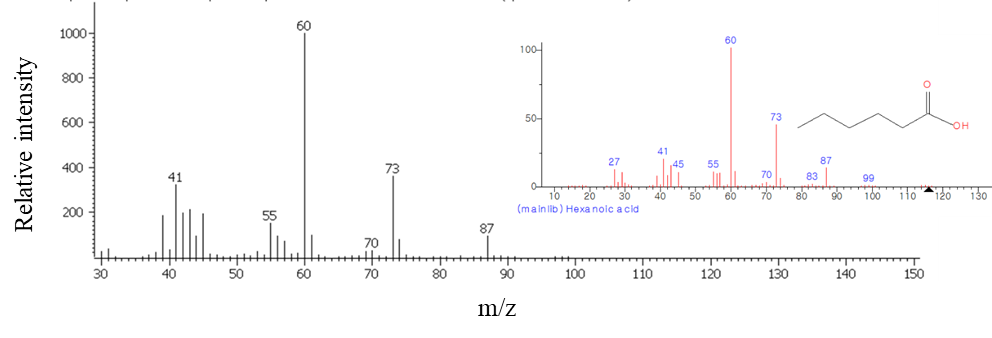


(d)


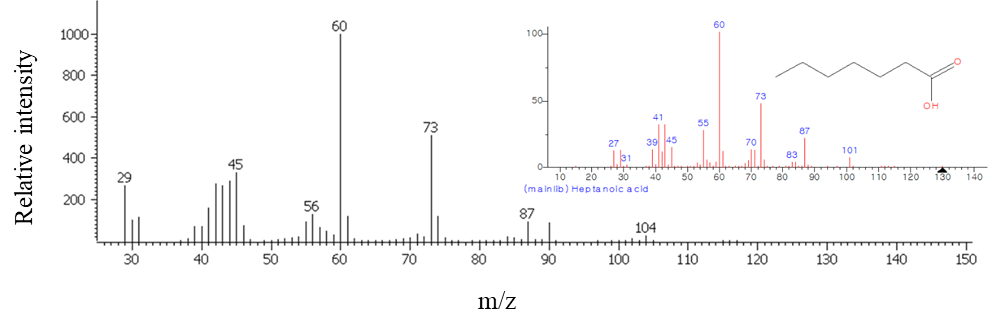


**Fig. S3.** Mass spectrum of CO_2_ in the head space of *Megasphaera hexanoica* with [1,2,3-^13^C_3_] propionate. The area ratio of the peaks at m/z 45 and 44 was 0.016. ^13^CO_2_ was rare.


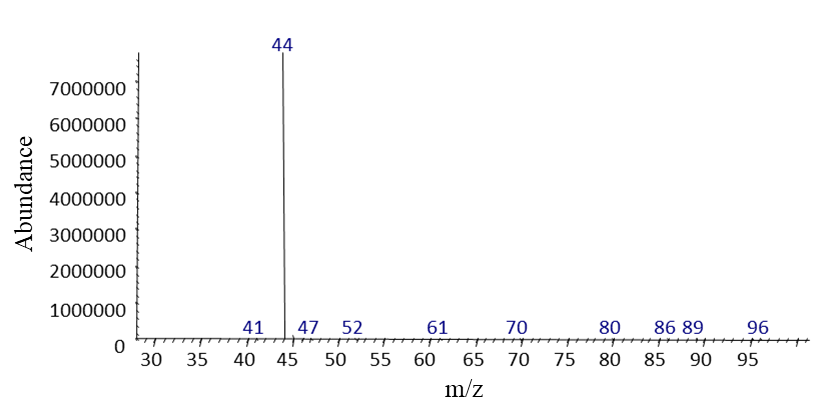


**Table S1**. Central composite experimental design matrix and experimental responses.

| Run | Real value level (g L^-1^) | | Coded value | | Heptanoic acid (g L^-1^) |
| --- | --- | --- | --- | --- | --- |
|  | Sodium acetate | Sodium propionate | X_1_ | X_2_ |  |
| 1 | 2.32 | 17.50 | -1 | -1 | 2.51 |
| 2 | 2.48 | 17.50 | 1 | -1 | 2.84 |
| 3 | 2.32 | 22.50 | -1 | 1 | 0.39 |
| 4 | 2.48 | 22.50 | 1 | 1 | 0.68 |
| 5 | 2.29 | 20.00 | -1.41 | 0 | 2.41 |
| 6 | 2.51 | 20.00 | 1.41 | 0 | 2.94 |
| 7 | 2.40 | 16.46 | 0 | -1.41 | 3.45 |
| 8 | 2.40 | 23.54 | 0 | 1.41 | 0.07 |
| 9 | 2.40 | 20.00 | 0 | 0 | 2.36 |
| 10 | 2.40 | 20.00 | 0 | 0 | 2.33 |
| 11 | 2.40 | 20.00 | 0 | 0 | 2.98 |
| 12 | 2.40 | 20.00 | 0 | 0 | 2.65 |
| 13 | 2.40 | 20.00 | 0 | 0 | 3.00 |

**Table S2.** Results of the statistical analysis of central composite experimental design for heptanoic acid production.

|  | Coefficient estimate | *t*-Value | *p*-Value |
| --- | --- | --- | --- |
| Intercept | 2.67 | 14.57 | <0.0001 |
| X_1_ | 0.17 | 1.17 | 0.2793 |
| X_2_ | -1.13 | -7.82 | 0.0001 |
| X_1_X_2_ | -0.01 | -0.05 | 0.9646 |
| X_1_^2^ | -0.17 | -1.03 | 0.3379 |
| X_2_^2^ | -0.60 | -3.89 | 0.0060 |
|  |  | F-Value | *p*-Value |
| Model |  | 15.59 | 0.0011 |
| Lack of fit |  | 2.50 | 0.1987 |

*R^2^* = 91.76%, *R^2^* (adjusted) = 85.87%

Heptanoic acid production (g L^-1^) = 2.67 – 1.13 X_2_ – 0.60 X_2_^2^  Equation S1

**Table S3.** Analysis of variance (ANOVA) for response surface quadratic model (Valeric acid).

| Source | DF | Sum of square | Mean square | *F* | *p* |
| --- | --- | --- | --- | --- | --- |
| Linear | 2 | 58.223 | 29.111 | 15.90 | 0.002 |
| Square | 2 | 61.152 | 30.576 | 16.70 | 0.002 |
| Interaction | 1 | 0.640 | 0.640 | 0.35 | 0.573 |
| RE | 7 | 12.816 | 1.831 |  |  |
| Lack of fit | 3 | 10.037 | 3.346 | 4.81 | 0.082 |
| Pure error | 4 | 2.780 | 0.695 |  |  |

**Table S4.** Analysis of variance (ANOVA) for response surface quadratic model (Heptanoic acid).

| Source | DF | Sum of square | Mean square | *F* | *p* |
| --- | --- | --- | --- | --- | --- |
| Linear | 2 | 10.518 | 5.259 | 31.24 | <0.001 |
| Square | 2 | 2.600 | 1.300 | 7.72 | 0.017 |
| Interaction | 1 | <0.001 | <0.001 | <0.01 | 0.965 |
| RE | 7 | 1.178 | 0.1683 |  |  |
| Lack of fit | 3 | 0.768 | 0.2561 | 2.50 | 0.199 |
| Pure error | 4 | 0.410 | 0.1025 |  |  |
